# Combined Pulse Electroporation – A Novel Strategy for Highly Efficient Transfection of Human and Mouse Cells

**DOI:** 10.1371/journal.pone.0009488

**Published:** 2010-03-02

**Authors:** Thorsten Stroh, Ulrike Erben, Anja A. Kühl, Martin Zeitz, Britta Siegmund

**Affiliations:** Charité Universitätsmedizin Berlin, Medizinische Klinik I, Berlin, Germany; Deutsches Krebsforschungszentrum, Germany

## Abstract

The type of a nucleic acid and the type of the cell to be transfected generally affect the efficiency of electroporation, the versatile method of choice for gene regulation studies or for recombinant protein expression. We here present a combined square pulse electroporation strategy to reproducibly and efficiently transfect eukaryotic cells. Cells suspended in a universal buffer system received an initial high voltage pulse that was continuously combined with a subsequent low voltage pulse with independently defined electric parameters of the effective field and the duration of each pulse. At comparable viable cell recoveries and transfection efficiencies of up to 95% of all cells, a wide variety of cells especially profited from this combined pulse strategy by high protein expression levels of individual cells after transfection. Long-term silencing of gene expression by transfected small interfering RNA was most likely due to the uptake of large nucleic acid amounts as shown by direct detection of fluorochromated small interfering RNA. The highly efficient combined pulse electroporation strategy enables for external regulation of the number of naked nucleic acid molecules taken up and can be easily adapted for cells considered difficult to transfect.

## Introduction

In the early 1980ies, upcoming recombinant techniques required efficient introduction of nucleic acids into eukaryotic cells. Various chemical, physical or virus-based transfection and transduction strategies were developed [1], [2]. Dependent on type and/or cell cycle state of the target cell population, these techniques might provide low transfer efficacies, be not suitable for non-adherent cells, require toxic reagents, give limited access to the nucleus, or are time-consuming [3], [4], [5], [6]. The application of strong electric fields to form pores within biological membranes was described as early as 1965 [7]. Ever since the first description of myeloma cell transfection [8], electroporation became the method of choice for efficient transient transfection for numerous types of eukaryotic cells. The so-called nucleofection combines electric pulses and chemical transduction to direct for the nucleus [9].

For electroporation in general, the strength of the electrical field applied and the pulse duration represent key parameters for effective compound delivery as well as for target cell viability [10], [11], [12]. Standard devices predominantly work with exponential decay pulses where a capacitor is charged to an initial voltage and is subsequently discharged through the cell suspension within a cuvette. Here, the time constant of individual pulses results from the capacity and overall resistance of the cell suspension. As for another type of electric pulses, square wave pulses rely on a constant electric field applied to the cell suspensions for a distinct time. Thus, creation of square wave pulses requires a circuit to terminate capacitor discharge after defined pulse duration. Compared to single pulses, a combination of membrane permeabilization and intracellular DNA transport by electrophoresis should increase transfection efficacies for DNA [13]. Studies addressing transfections could demonstrate an improvement of pulse combinations in contrast to single pulse strategies in vivo [14], [15]. However, the experimental situation in tissues differs significantly from conditions in vitro. Thus results not easily transfer from one system to the other. In previous in vitro studies, a combination of high voltage (HV) and low voltage (LV) pulses was advantageous if the pulses were separated by gaps of 100 µs to 20 ms [16], [17]. Here we present an electroporation strategy with continuous combined HV and LV square pulses where, using the modular ELPorator 1000 device, the electric parameter of the effective field and the duration of each pulse can be defined individually. This method allows for highly efficient transfection of DNA or siRNA into isolated primary cell populations or within mixed cell suspensions as well as into other cell populations that are considered difficult to transfect. This combined HV/LV strategy not only enables the transfer of high amounts of molecules per cell but also allows to externally regulating the amount of transfected molecules.

## Methods

### Ethics Statement, Primary Cells and Cell Lines

Six- to eight-week old female C57BL/10 mice were from the Charité - Forschungseinrichtungen für Experimentelle Medizin (Berlin, Germany) and the regional animal study committee of Berlin, Germany approved the animal protocols. Whole blood samples from healthy donors were obtained with written consent and with the approval of the local ethics committee of the Charité – Universitätsmedizin Berlin. Peripheral blood mononuclear cells (PBMC) were prepared from the heparinized samples by Ficoll density gradient centrifugation (GE Healthcare, Munich, Germany). For staining of subpopulations directly labeled monoclonal antibodies specific for CD4/clone 13B8.2, CD8/clone BW135/80, CD14/clone MΦP9 or CD19/clone HIB19 (BD Biosciences, Heidelberg, Germany) were used. The murine cell lines 3T3L1 (ATCC CL-173) and J558L (ATCC TIB-6) were from the American Type Culture Collection (Bethesda, MD); the human cell lines K562 (ACC 10) and U937 (ACC 5) from the *Deutsche Sammlung von Mikroorganismen und Zellkulturen* (Braunschweig, Germany).

### Isolation of Mouse Preadipocytes and Cell Culture

Murine preadipocytes were prepared and propagated up to ten passages as previously described [18]. In brief, excised mesenteric adipose tissue was minced and washed in HBSS. Tissue was digested within 25 min at 37°C in HBSS containing 1.5 mg/ml collagenase II, 3.5% BSA and 550 µM glucose. The cell suspension was subsequently mashed through a 100 µm-nylon net (BD Biosciences), sedimented at 200 *g* for 10 min and washed twice in DMEM/HAMS F-12 containing 10% FCS and penicillin/streptomycin (100 units/ml each). After 24 h incubation in 48-well plates (5×10^5^ cells/well), non-adherent cells were discarded and adherent cell layers were propagated to establish preadipocyte cell lines that were used up to the 10th passage. Cells of the murine preadipocyte cell line 3T3L1 (ATCC CL-173) were propagated in DMEM containing 10% FCS and penicillin/streptomycin (100 units/ml each). The potential to give rise to fully differentiated adipocytes from all preadipocyte cell lines used in this study was confirmed by lipid droplet staining with Oil red O after incubation with insulin, dexamethasone, and 3-isobutyl-1-methylxanthine as described previously [19].

### Electroporation of Eukaryotic Cells by the ELPorator 1000 Device

**Figure 1:**
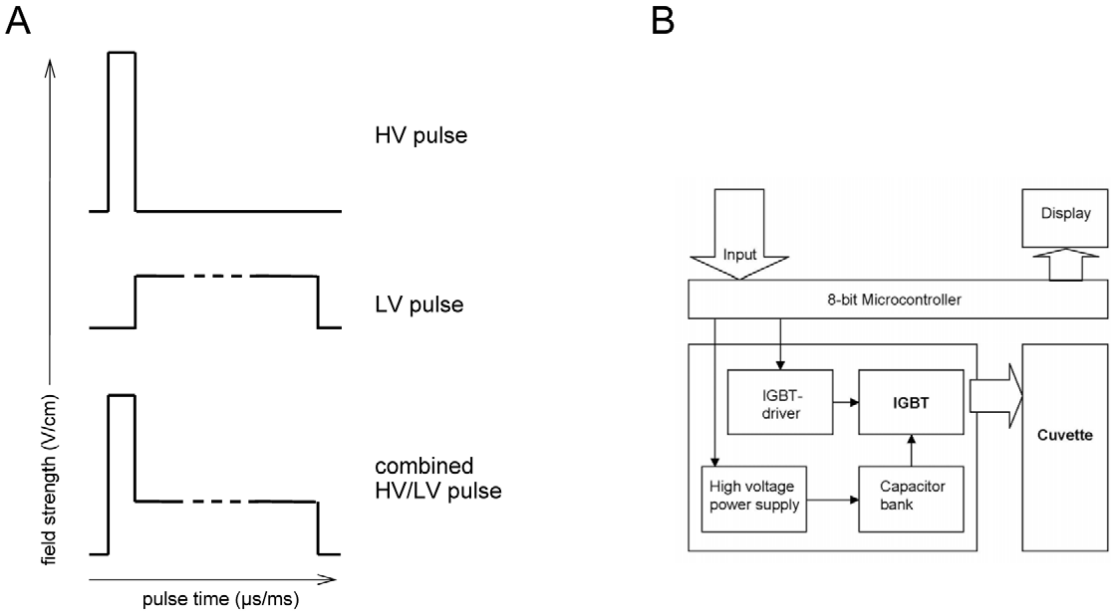
Definition of square wave pulses for eukaryotic cell transfection generated by the ELPorator 1000 device. (A) The ELPorator 1000 device consisting of two physically independent but electrically interconnectable modules. Module 1 produces single HV square wave pulse at a maximum field strength of 5 kV/cm for 1–999 µs. Module 2 provides a single LV square wave pulse with up to 1.5 kV/cm in the range of milliseconds. Combination of HV and LV square wave pulses forms an uninterrupted combined pulse. Preferentially, a LV pulse follows a primary HV pulse. Within the ranges noted above, parameters of single or combined pulses can be defined individually. (B) Flow chart of input and output parameters within the electronic components of the individual modules.

The complete ELPorator 1000 device consists of two modules, generating independent square wave pulses that can be connected electrically to generate a continuous combined pulse (Fig. 1A). Module 1 provides voltages of 0–1.0 kV at a pulse length of 0–999 µs; module 2 delivers 0–0.3 kV at a pulse length of 0–99 ms. Both modules consist of a capacitor bank charged by a high voltage power supply. Different output field strengths result from different dimensions of the capacitor bank in each module (module 1: 110 µF; module 2: 940 µF). Electric charge stored in the capacitor bank is delivered to the cuvette via an Insulated Gate Bipolar Transistor (IGBT; Infineon, Munich, Germany). This high performance semiconductor handles collector/emitter voltages up to 1.2 kV, pulses collector currents up to 84 A and is controlled via a Gate Resistor by an IGBT Driver (Avago Technologies, San Jose, CA) which also enables for switching times in the nanosecond range. An 8-bit Reduced Instruction Set Computing Microcontroller (Atmel, San Jose, CA) controls the IGBT driver. Microcontroller programs in assembler language allow for user defined pulse voltages and pulse length as input parameters (Fig. 1B). The capacitors connected in series to resist voltages up to 1.2 kV and the IGBT are designed to handle overall high voltages and currents. For safety reasons, a shunt resistor adjusts the current flow through the cuvette. At current flows above 60 A, a signal to the microcontroller activates the driver circuit and an additional relay to immediately interrupt the pulse and to avoid damages from an electric arc (Fig. S1). For combined HV/LV pulses, modules 1 and 2 can be connected in parallel. Diodes at the outputs prevent current overflow from module 1 into the lower charged capacitor bank of module 2.

### Transfection with Plasmid DNA

The expression vector pEGFP-N1 (GeneBank Accession #U55762; 4,651 bp; Clontech, Mountain View, CA) was used to assess plasmid transfection efficiencies. Plasmid preparations from a standard anion-exchange method according to the manufacturer's instructions (Macherey-Nagel, Düren, Germany) typically contained >95% covalently closed circular DNA. Cell lines or murine preadipocytes from cell culture or freshly prepared PBMC (1–3×10^6^) were resuspended in 100 µl electroporation buffer containing 90 mM phosphate buffer, pH 7.2, 10 mM MgCl_2_ and 50 mM glucose before 4 µg pEGFP-N1 were added. Using an electroporation cuvette (2 mm gap; Lonza, Cologne, Germany) in the ELPorator 1000 device, cells were subjected to a single HV or LV, or to continuously combined HV/LV pulses (Fig. 1A). Alternatively, 1×10^6^ 3T3L1 cells or primary murine preadipocytes were transfected in a Nucleofector I device (Lonza) with the Cell Line V Nucleofector Kit and the T-030 program according to the manufacturer's protocol.

Immediately after pulse application, cells were transferred into the respective pre-warmed standard culture medium containing 10% FCS and were incubated at 37°C for 24 h. Cells were harvested and vital cell recovery assessed by trypan blue exclusion. Transfection efficiencies as estimated by percentages of EGFP^+^ cells and individual mean fluorescence intensity (MFI) values were analyzed by flow cytometry using a FACSCanto II™ device and the FACSDiva™ software (version 5.0.2; BD Biosciences, Heidelberg, Germany).

### Quantification of Glyceraldehyde-3 Phosphate Dehydrogenase mRNA Using Plasmid Standard with a Cloned Glyceraldehyde-3 Phosphate Dehydrogenase Fragment

A fragment of murine glyceraldehyde-3 phosphate dehydrogenase (GAPDH) was amplified from cDNA of murine preadipocytes by GAPDH-specific PCR using the primer pair 5′–CATCCTGCACCACCAACTGC–3′/5′–ACGCCACAGCTTTCCAGAGG–3′ (TIB Molbiol, Berlin, Germany). The sequence cloned into the vector pCR2.1 (Invitrogen, Karlsruhe, Germany) was verified (Seqlab, Göttingen, Germany) to GenBank accession no. NM_008084. The plasmid copy number of linearized DNA was calculated from the concentration as determined by spectrophotometry using a NanoDrop ND-1000 device (NanoDrop Technologies, Wilmington, NC) and the molecular weight of the plasmid DNA. Single-use aliquots were stored at −80°C. The PCR mixture contained 2 µl plasmid standard, 2.5 pmol of sense and antisense primers and the SYBR Green PCR Master Mix (Applied Biosystems, Foster City, CA) in a total volume of 12.5 µl. Polymerase activation and target amplification were performed using the following protocol: 10 min at 95°C, 35 cycles consisting of 15 sec at 95°C followed by 60 sec at 62°C using a StepOne Plus™ Real-Time PCR System and the instruments software (Applied Biosystems). For a quantification standard curve, the coefficient of determination of a series of log10 dilutions from 1×10^8^ to 1×10^2^ GAPDH copies had to be at least 0.997. Copy numbers of GAPDH mRNA in experimental samples were calculated from the cell count as determined by trypan blue exclusion and individual crossing points from concomitant PCR.

### Transfection with Small Interfering RNA

Preadipocytes (1×10^6^) were resuspended in 100 µl electroporation buffer before GAPDH-specific (Silencer® Cy™3; Ambion, Austin, TX) or Toll-like receptor (TLR) 4-specific (sense: 5′-GGAAGUUCACAUAGCUGAAdTdT-3′, antisense: 5′-UUCAGCUAUGUGAACUUCCdGdG-3′; Qiagen, Hilden, Germany) or control siRNA (sense: 5′-UUCUCCGAACGUGUCACGUdTdT-3′, antisense: 5′-ACGUGACACGUUCGGAGAAdTdT-3′; Qiagen, Hilden, Germany) was added and subjected to single or continuously combined pulses as described above. As to determine the efficacy of siRNA uptake and function, knockdown of GAPDH expression was assessed from reversely transcribed mRNA of defined cell numbers by quantitative PCR using the plasmid standard and SYBR-Green. The effects of siRNA on GAPDH expression was determined from reversely transcribed mRNA that corresponds to 410 cells. Intracellular presence of Cy3-labeled GAPDH-specific siRNA was directly monitored by flow cytometry in propidium iodide negative cells. One day after transfection with the TLR4-specific siRNA, cells were stimulated with 1 µg/ml LPS the specific TLR4 ligand [20]or with 200 nM lactyl-tetra-diaminopimelic acid (LT-DAP) the specific ligand for the nucleotide oligomerization domain-1 [21]. After another 24 h, IL-6 production was determined from the cell culture supernatants by Cytometric Bead Array (BD Biosciences, Heidelberg, Germany) according to the manufacturer's protocol.

### Statistical Analysis

Significances were determined by two-sided t-tests using Prism 4 software for Windows (GraphPad Software, San Diego, CA).

## Results

### Permanent Cell Lines and Primary Adherent Cells as Exemplified for Preadipocytes Can Be Effectively Transfected by Combined HV/LV Pulses

**Figure 2:**
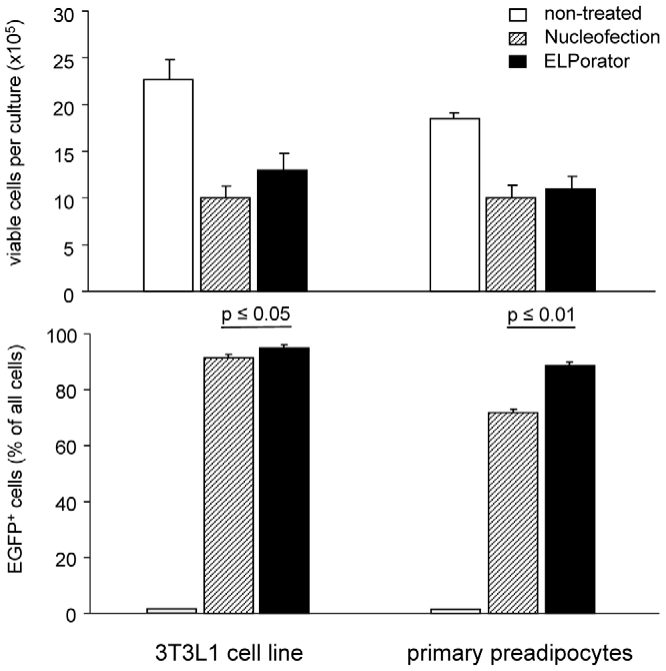
Transfection efficiency for adherent cells achieved by nucleofection compared to the ELPorator 1000 device. For transfection of 1×10^6^ 3T3L1 cells or preadipocytes from the mesenteric fat with 2 µg pEGFP-N1, a pulse combined of a HV pulse of 3 kV/cm for 400 µs followed by a LV pulse of 1.5 kV/cm for 5 ms was applied by the ELPorator 1000 device. Alternatively, cells were transfected using the Nucleofector Kit V and program T-030 in a device for nucleofection or remained untreated. After 24 h, cell recovery and viability were assessed by trypan blue exclusion. Transfection efficiency was determined by flow cytometry as percentage of EGFP^+^ cells. Mean values ± SEM; n = 5.

Initial experiments established baseline standard conditions for the application of combined HV/LV pulses compared to standard transfection techniques. Independently varied parameters for single HV and LV pulses or combined HV/LV pulses revealed optimum transfection for 3T3L1 cells or primary murine preadipocytes with an initial HV pulse of 3 kV/cm for 400 µs followed without a gap by a LV pulse of 1.5 kV/cm for 5 ms. Cell viability and transfection efficiency were compared with nucleofection as an established electroporation method. Generally, electroporation reduced viable cell numbers recovered after 24 h of culture by about half compared to non-treated control cells (Fig. 2, upper panel). Under conditions recommended for nucleofection of 3T3L1 cells, transfection efficiencies as determined by the percentage of EGPF^+^ cells were higher for the 3T3L1 cell line (91.5±1.3%) than for primary preadipocytes (71.7±2.8%) (Fig. 2, lower panel). Using optimized combined HV/LV pulses provided by the ELPorator 1000 device, both cell types were transfected comparably (3T3L1: 95.0±1.3%; primary: 88.6±2.3%) and transfection efficiencies were significantly higher than by nucleofection (3T3L1: p<0.05; primary: p<0.01). These high percentages of transfected cells allowed e.g. for immediate further experiments without enrichment procedures related to the recombinant protein.

### Transfection Efficiency of Electroporation Strictly Depends Upon Pulse Composition and DNA Amounts

**Figure 3:**
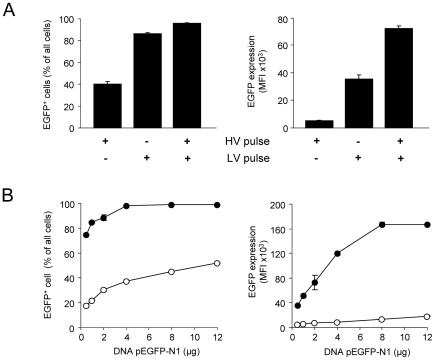
Impact of pulse composition and plasmid DNA amount on the transfection efficiency for primary murine preadipocytes. (A) 3T3L1 cells were transfected with 2 µg pEGFP-N1 using the ELPorator 1000 device for single HV or LV pulses or for a combined HV/LV pulse (HV: 3 kV/cm, 400 µs; LV: 1.5 kV/cm, 5 ms). Efficiency was estimated by flow cytometry from the percentages of EGFP^+^ cells and from individual EGFP expression levels after 24 h. Mean values ± SEM, n = 2. (B) Primary murine preadipocytes were transfected with different amounts of pEGFP-N1 vector using a single HV pulse (3 kV/cm, 400 µs; white circles) or a combined HV/LV pulse (3 kV/cm, 400 µs and 1.5 kV/cm, 5 ms; black circles). Transfection efficiency was determined from the percentages of EGFP^+^ cells and EGFP expression levels after 24 h. Mean values ± SEM; n = 3.

Cell-type specific effects of different pulse parameters were assessed by single HV, single LV or combined HV/LV pulses applied to 3T3L1 cells using the ELPorator 1000 device. A single HV pulse gave rise to about 40% EGFP^+^ cells (Fig. 3A). Remarkably, a single LV pulse or a combined HV/LV pulse resulted in significantly higher transfection efficiencies of approximately 90% EGFP^+^ cells. At first sight, these cells with a high cytoplasma/nucleus ratio did not seem to benefit from the combined pulse strategy for transfection with plasmid DNA. If focusing on cellular expression levels of the recombinant protein, a single HV pulse again was inferior for transfection compared to the other strategies. In contrast to the findings with percentages of EGFP^+^ cells, mean fluorescence intensity (MFI) values determined by flow cytometry to assess intracellular fluorescent protein expression levels were significantly higher with the combined HV/LV pulse compared to the single LV pulse. These findings underline the importance of externally modifiable pulse properties to optimize uptake and define intracellular fate of given DNA amounts.

Next, we asked how DNA amounts affect transfection efficiency (Fig. 3B). Despite a slight increase from 30.4±0.9% EGFP^+^ cells with 2 µg DNA to 52.0±0.5% with 12 µg DNA, recombinant protein expression levels remained unchanged if increasing amounts of plasmid DNA were used with a single HV pulse for electroporation. With the combined HV/LV pulse strategy, 4 µg DNA sufficiently transfected all preadipocytes and this plateau level was not altered by increasing amounts of DNA, as determined with up to 12 µg plasmid DNA. Comparable to varied pulse parameters, EGFP expression levels increased with DNA amounts up to 8 µg until a MFI plateau level of 167.5×10^3^±3.5×10^3^ arbitrary units was reached. This DNA dose dependency indicated that transfection efficiencies to be obtained employing the ELPorator 1000 device could be modulated by the electrical conditions as well as by the plasmid DNA amount to be transfected.

### Combined Continuous HV/LV Pulse Strategy Allows for Efficient Transfection of Suspension Cell Lines

The murine myeloma cell line J588L, the human erythromyeloblastoid leukemia cell line K562 and the human histiocytic lymphoma cell line U937, all suspension cells frequently used for recombinant protein expression or to study phenomena related to malignant transformation or cellular differentiation were treated with a combined HV/LV pulse to transfect 4 µg standard plasmid DNA. These cells were efficiently transfected with a uniform set of electric parameters for a combined HV/LV pulse (Table 1). More than 90% of the J558L and K562 cells were EGFP^+^ with high cellular fluorescent protein expression levels as shown by MFI values of about 10^5^ arbitrary units. Of the usually difficult to transfect U937 cells, 79.1±0.5% were EGFP^+^ with intermediate EGFP expression. The cell loss of about a third of the J558L or K562 cells and of about half of the U937 cells after application of a combined HV/LV pulse with the ELPorator 1000 device, was recompensed by high transfection efficiencies. Overall recovery of vital cells after transfection of these three suspension cell lines was higher applying single HV pulses, but accompanied by lower MFI values. With exception of the U937 cell line where transfection efficiency as determined by percentages of EGFP^+^ cells was nearly doubled (45.5 vs. 79.1; MFI: 9.4×10^3^ vs. 42.1×10^3^) (Fig. S2), a combined HV/LV did not markedly affect the number of transfected cells compared to a single HV pulse. As shown for adherent cells (Fig. 3), the key benefit of the combined pulse strategy was an increase in recombinant intracellular fluorescent protein levels. For example, applying a HV/LV pulse to J558L myeloma cells, the expression level of EGFP was about four times higher in comparison to levels that could be reached if only using a single HV pulse (MFI: 95.5.x10^3^ vs. 29.6×10^3^) (Fig. S2).

**Table 1:** Transfection efficiencies and viable cell recovery in suspension cell lines.

Cell line	Transfection efficiency		Viable cell recovery (×10^5^)	N
	EGFP^+^ cells (% of all cells)	MFI (x10^3^)		
J558L	92.2±0.5	95.5±10.0	6.4±0.8	4
K562	95.0±0.6	107.2±10.4	7.3±0.8	3
U937	79.1±0.5	42.1±2.7	4.3±0.5	4

Suspension cells from standard cultures (1×10^6^) were transfected with 4 µg pEGFP-N1 vector using a combined HV/LV pulse (3 kV/cm, 100 µs and 750 V/cm, 10 ms) applied by the ELPorator 1000 device. After 24 h, transfection efficiency was determined by flow cytometry from the percentages of EGFP^+^ cells and from individual EGFP expression levels. Viable cell recovery was assessed by trypan blue exclusion. Mean values ± SEM.

### Immune Cells in Mixed Peripheral Blood Mononuclear Cell Preparations Are Efficiently and Subtype-Dependently Transfected by a Strategy of Combined HV/LV Pulses

**Figure 4:**
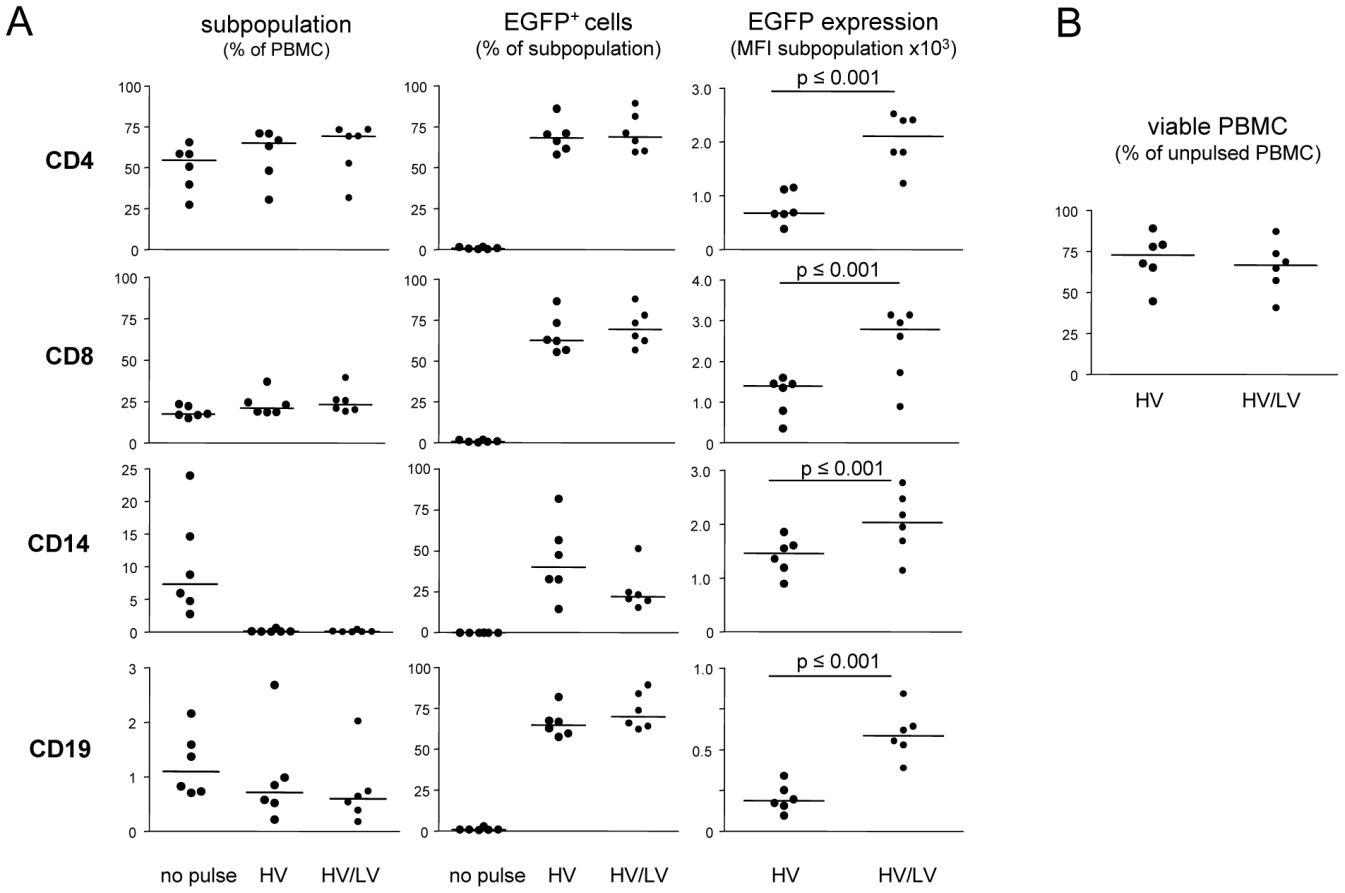
Transfection efficiency of defined lymphocytic and monocytic cell subpopulations within mixed human peripheral blood mononuclear cells. Freshly prepared human PBMC (3×10^6^) were transfected with 4 µg pEGFP-N1. A single HV pulse of 4.5 kV/cm for 400 µs or combined with a LV pulse of 0.75 kV/cm for 3 ms was applied by the ELPorator 1000 device. Non-treated PBMC served as controls. (A) Cells were incubated with directly labeled monoclonal antibodies and overall distribution of the subpopulations within mixed PBMC preparations, percentages of EGFP^+^ cells, and EGFP expression levels within the individual populations were determined by multi-color flow cytometry after 24 h. Mean values from triplicate determinations; n = 6. (B) Viability of treated cells was assessed after 24 h by propidium iodide staining. Mean values from triplicate determinations; n = 6.

Freshly isolated PBMC comprise interesting cell populations e.g. to study immune cell-mediated mechanisms against the background of defined major histocompatibility complex molecules. We asked whether the main subpopulations of T and B lymphocytes or monocytes within mixed PBMC preparations were efficiently recovered and transfected for recombinant protein expression if treated with single HV or combined HV/LV pulses. Recovery of subpopulations after transfection was estimated by specific staining and flow cytometry analysis (Fig. 4A). Percentages of CD4^+^ T cells were marginally increased after HV or combined HV/LV pulses, while the proportions of CD8^+^ T cells were not affected by transfection. CD19^+^ cells showed a distinct decrease, identifying B cells to be more sensitive to transfection conditions applied. Transfection efficiency as estimated by percentages of EGFP expressing cells was comparable in all lymphocyte subpopulations. A combined HV/LV transfection condition slightly increased the transfection efficiency of CD8^+^ and CD19^+^ cells compared to the single pulse but had no effect on CD4^+^ cells. Again, after applying a combined HV/LV pulse the cellular EGFP expression level was significantly higher in all subpopulations investigated, while the additional LV pulse did not reduce overall cell recovery (Fig. 4B). CD14^+^ cells within the mixed PBMC populations proved to be very sensitive to HV or HV/LV pulses. Although under the conditions described, up to 98% of the CD14^+^ cells were depleted, however the surviving monocytic cells were sufficiently transfected. Thus, efficient transfection of freshly prepared PBMC might compensate for the need of further time-consuming procedures to isolate specific subpopulations if subsequent read-out procedures allow for the discrimination of immune cell subpopulations. Reasonable transfection efficiencies render this an interesting approach even for primary CD14^+^ cells if they can be further distinguished from other of PMBC subpopulations.

### Transfection with Combined HV/LV Pulses Grants Long-Lasting Gene Knockdown by Efficient Delivery of siRNA

**Figure 5:**
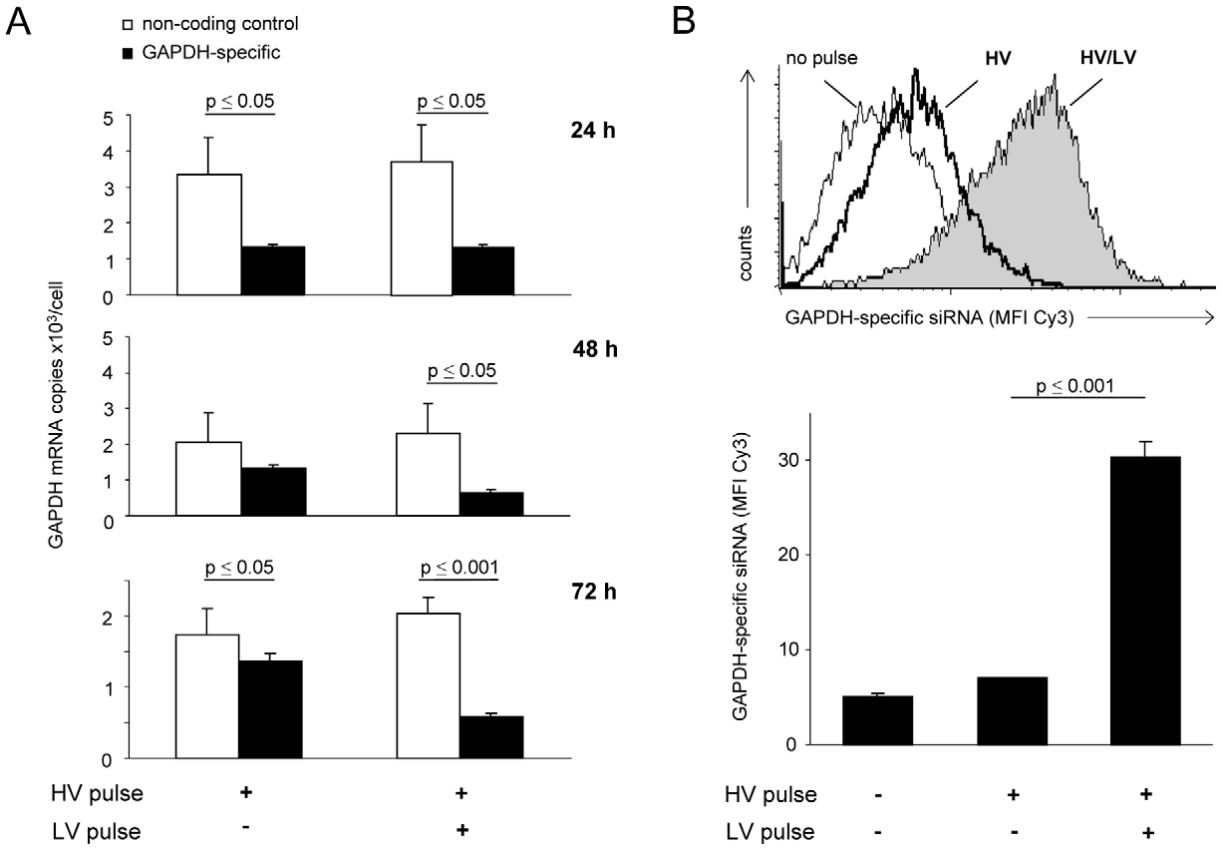
Long-term outcome of transfection of primary preadipocytes with GAPDH-specific siRNA. Primary preadipocytes were transfected with 120 pmol Cy3-labeled GAPDH-specific siRNA or a non-coding siRNA for control using a single HV pulse (3 kV/cm, 400 µs) or a combined HV/LV pulse (3 kV/cm, 400 µs and 1.5 kV/cm, 5 ms) produced by the ELPorator 1000 device. (A) After 24, 48 and 72 h, copy numbers of GAPDH-specific mRNA were determined by quantitative PCR in comparison to a cloned plasmid standard. Mean values ± SEM; n = 6. (B) As directly assessed by flow cytometry 5 h after transfection, uptake of Cy3-labeled GAPDH-specific siRNA by preadipocytes was determined via the MFI. Representative histogram and mean values ± SEM; n = 4.

**Figure 6:**
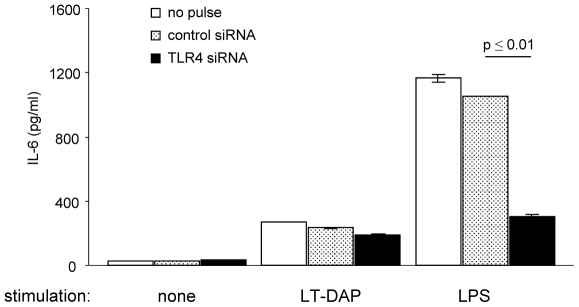
Functionality of siRNA in transfected cells. Primary preadipocytes were transfected with 100 pmol TLR4-specific siRNA or non-coding control siRNA using the combined HV/LV pulse strategy (3 kV/cm, 400 µs; 1.5 kV/cm, 5 ms). One day after transfection, cultures received 1 µg/ml LPS or 200 nM LT-DAP for additional 24 h before IL-6 production was determined from the cell culture supernatants. Bars represent the mean of n = 3 ± SEM.

As for a model system for knockdown with the siRNA molecules, primary murine preadipocytes were transfected with 120 pmol siRNA specific for GAPDH by a single HV pulse or a combined HV/LV pulse. Independent of the pulse strategy, GAPDH-specific siRNA revealed reduced copy numbers of GAPDH mRNA compared to a non-coding control siRNA after 24 h (Fig. 5A, upper panel) and 48 h (Fig. 5A, middle panel). Even after 72 h, copy numbers of GAPDH-specific mRNA remained significantly reduced (Fig. 5A, lower panel). Here in a time span interesting for transient but long-term knockdown, the combined HV/LV pulse (p<0.001) was more efficient than the single HV pulse (p<0.05). To correlate the efficiency of siRNA uptake directly with the efficiency of GAPDH-specific knockdown, transfected GAPDH-specific siRNA was visualized by flow cytometry via a fluorescent Cy3 label (Fig. 5B). Five hours after transfection with GAPDH-specific siRNA using a single HV pulse, MFI values were only slightly higher than for non-transfected preadipocytes. However, the amount of cell-associated Cy3 was significantly increased by about 7-fold if labeled siRNA was delivered using the combined HV/LV pulse strategy. As for an additional functional readout, preadipocytes transfected with a TLR4-specific siRNA showed significantly reduced IL-6 secretion upon stimulation with LPS compared to the controls. Emphasizing the specificity of the TLR4 knock-down, stimulation with the specific ligand for nucleotide oligomerization domain-1, a related intracellular receptor, was not altered by transfection with TLR4-specific siRNA compared to cells that received the control siRNA (Fig. 6). These findings not only underline the advantages of the combined square pulse strategy, technically realized with the ELPorator 1000 device, but also confirmed the concept that efficiency and duration of a specific knockdown directly depend on the amount of intracellular siRNA.

## Discussion

The modular ELPorator 1000 device grants controlled, external presetting of field strength and pulse length of square pulses, basic electric parameters that define the transfection efficiency for eukaryotic cells. In addition, this configuration allows uninterrupted combined HV and LV pulses that help to optimize conditions with regard to the nature of the cell populations and of the nucleic acids to be transfected. This individualized approach facilitates not only the transfection of a wide variety of cell types, but results in profound and long-lasting gene suppression after siRNA transfection. As for an established technique to efficiently transfect eukaryotic cells with DNA, nucleofection represents a variant of electroporation providing fixed electrical conditions in combination with specific buffer compositions [2], [9], [22], [23]. Transfection efficiencies improved under optimized conditions applying combined HV/LV square pulses using a standard universal phosphate buffer system as shown for preadipocytes (Fig. 2). How might combined HV/LV square pulses become advantageous for distinct cell populations? The high field strength of short HV pulses is thought to be sufficient to create pores that overall increase cell membrane permeability for large molecules [24], [25]. A LV pulse is considered to maintain pores without negative effects on the cell integrity for a given prolonged time, and thus allows for an electrophoretic transport of electrically charged molecules like nucleic acids [13]. Two earlier studies addressed the question whether joining these phenomena in a double pulse consisting of a HV pulse and a LV pulse is superior to single pulse strategies for eukaryotic cell transfection. Although ambiguous in the outcomes, a benefit of the double pulse strategies that included defined breaks between the HV and the LV square pulse seemed to be limited to experimental settings with low DNA amounts [16], [17]. We here relied on combined pulses where the LV pulse immediately followed the HV square pulse in terms of downsizing the field strength without interrupting the application of the electric field itself (Fig. 1). Using this approach, our study emphasizes the necessity to individualize and to control electrical conditions as well as to adapt the parameters along cell morphology and physiology, rather than the buffer conditions during electroporation. For cells with high cytoplasm/nucleus ratios, a single LV pulse was more efficient than a single HV pulse in transfecting e.g. primary preadipocytes with plasmid DNA. The combined HV/LV pulse strategy again significantly increased the transfection efficacy as determined by the percentage of transfected cells and by the expression levels of the recombinant protein in individual cells (Fig. 3). Having low cytoplasm/nucleus ratios like lymphocytes or suspension cells of tumor cell lines, transfection efficiencies using standard nucleofection conditions for total PBMC preparations were found to be in the range of 30–70% of all cells [26]. In our hands, the main proportion of the cells was transfected with given DNA amounts using a short single HV pulse. PBMC profited from a combined HV/LV pulse strategy showing enhanced individual expression levels of the recombinant protein (Fig. 4). We specifically did not aim to prove superiority to other electroporation methods but to clearly comprehend experimental conditions for the transfection of different cells using a combined pulse electroporation strategy.

Emphasizing that the efficient uptake of large amounts of nucleic acids under the field conditions of combined HV/LV pulses provided by the ELPorator 1000 device and not the promotor strength alone was responsible for the high expression levels, cells transfected with a combined HV/LV pulse contained significant amounts of fluorochrome-labeled siRNA as directly quantified by flow cytometry (Fig. 5A). Due to its small size, siRNA effectively enters eukaryotic cells upon chemical transfection, but inclusion within cytoplasmic vesicles might render siRNA inaccessible for down-regulation of gene expression [27], [28]. After electroporation using the combined HV/LV pulse strategy, no vesicle formation was observed and the transfected specific siRNA significantly suppressed model gene expression for up to 72 h (Figs 5 and 6); a time span suitable for experimental approaches that include specific knockdown.

Thus, with independently controllable but combined HV/LV square pulses efficient transfection of eukaryotic cells with plasmid DNA or siRNA can be easily established for cells where no standardized protocols exist so far, simply by modifying field strength and pulse length of the HV as well as a potential subsequent LV pulse using an universal buffer system for electroporation. This cell type-dependent rational approach provides additional insights to physical prerequisites for optimum transfection and typically results in high transfection efficiencies accompanied by good cell viability. Since our technical solution allows profound and long-lasting gene suppression after transfection of specific siRNA, we anticipate its use for a broad spectrum of normally hard-to-transfect cell populations including primary cells, and for a variety of siRNA applications.

## Supporting Information

Figure S1 Composition of the ELPorator 1000 device. Electronic composition of the output stage of the HV module. MC, microcontroller; OC, optical coupler; RG, gate resistor; T, transistor; RS, shunt resistor, D, diode; Rel, relay; C, capacitor (upper panel). Picture shows the complete ELPorator 1000 system (lower panel). Outputs of both modules are parallel connected via internal diodes to prevent current flow from one module to the other. The signal to trigger the LV module is generated by the microcontroller of the HV module and sent via a control cable at the backside of the module.
(0.58 MB TIF)

Figure S2 Transfection efficiencies for plasmid DNA to suspension cells after applying a single HV or a combined HV/LV pulse. Suspension cells from standard cultures (1×10^6^) were transfected with 4 µg pEGFP N1 vector using a single HV pulse (3 kV/cm, 400 µs; bold line) or a combined HV/LV pulse (3 kV/cm, 100 µs and 750 V/cm, 10 ms; gray area) applied by the ELPorator 1000 device. After 24 h, the transfection efficiency was determined by flow cytometry from the percentages of EGFP^+^ cells and from individual EGFP expression levels according to the MFI values. Light lines show the respective negative control. Viable cell recovery was assessed by trypan blue exclusion. Values given in individual columns are: EGFP^+^ cells as percentage of all cells; MFI x10^3^; viable cell recovery x10^5^. Data are representative for at least three comparable experiments done in triplicates.
(0.15 MB TIF)
