# Supplementary figures and images for: Combined Pulse Electroporation – A Novel Strategy for Highly Efficient Transfection of Human and Mouse Cells
Source: PLoS One. 2010 Mar 2;5(3):e9488. doi: 10.1371/journal.pone.0009488 (PMC2830457; doi:10.1371/journal.pone.0009488)

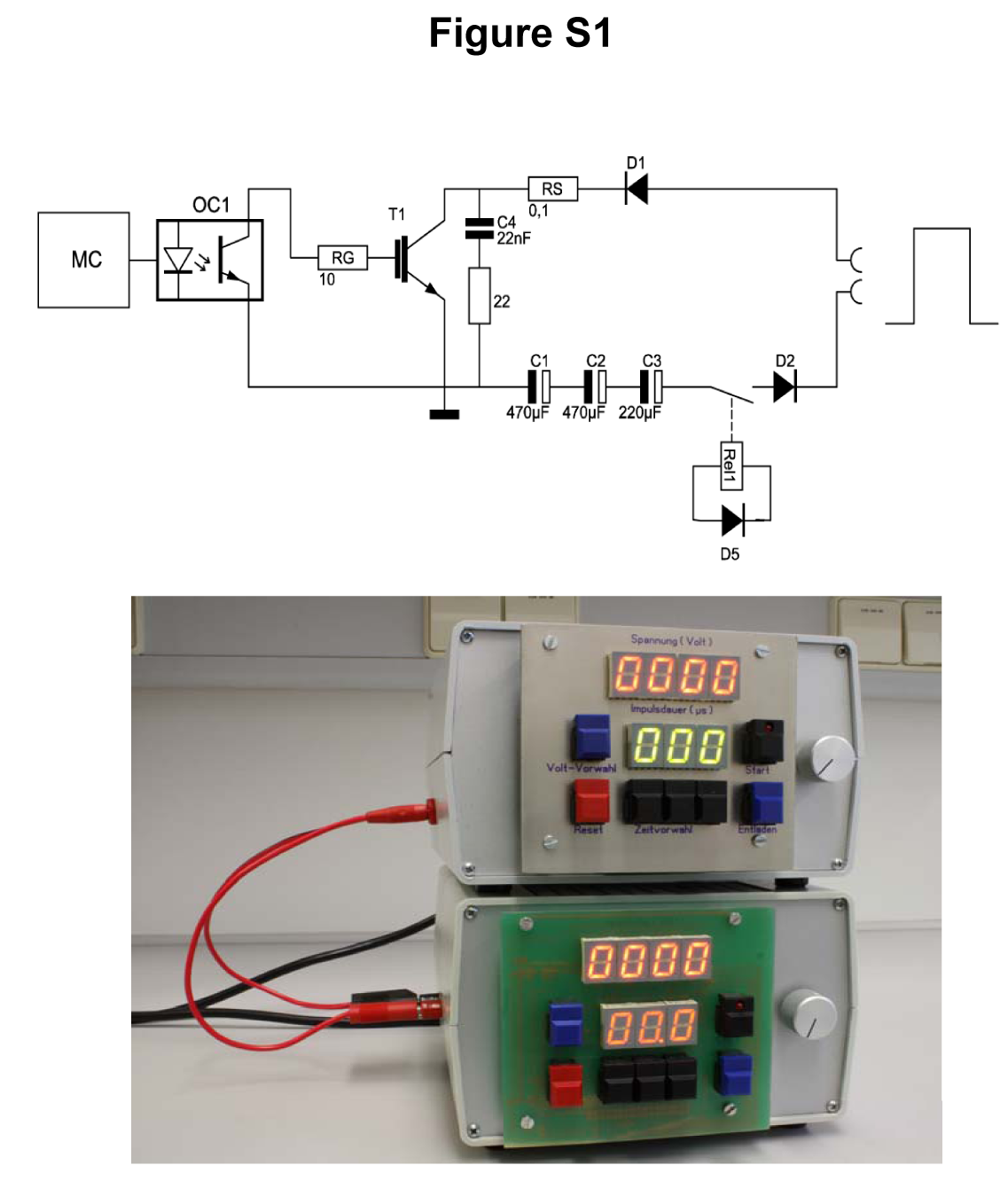

Supplement: Figure S1 — Composition of the ELPorator 1000 device. Electronic composition of the output stage of the HV module. MC, microcontroller; OC, optical coupler; RG, gate resistor; T, transistor; RS, shunt resistor, D, diode; Rel, relay; C, capacitor (upper panel). Picture shows the complete ELPorator 1000 system (lower panel). Outputs of both modules are parallel connected via internal diodes to prevent current flow from one module to the other. The signal to trigger the LV module is generated by the microcontroller of the HV module and sent via a control cable at the backside of the module. (0.58 MB TIF) [file pone.0009488.s001.tif]

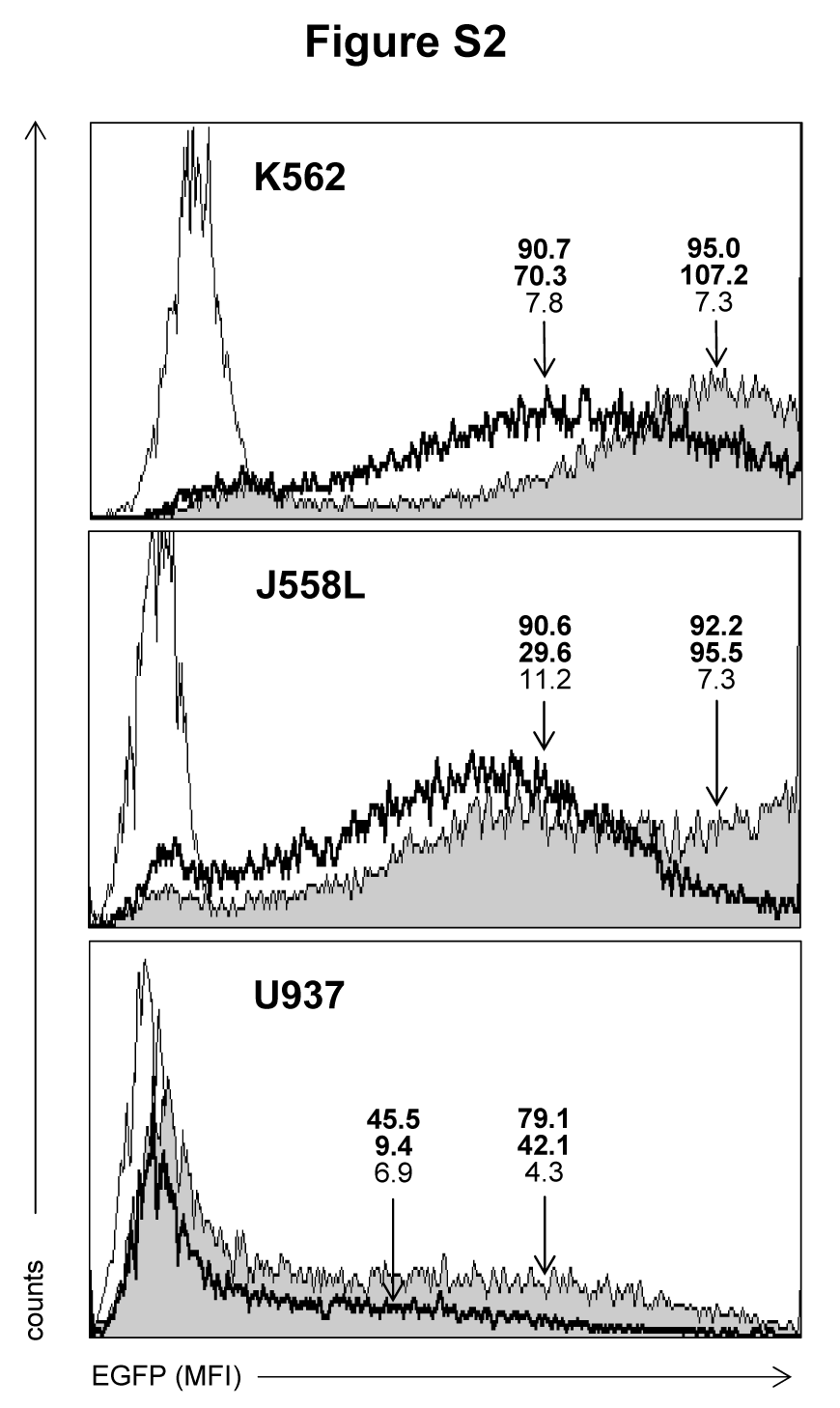

Supplement: Figure S2 — Transfection efficiencies for plasmid DNA to suspension cells after applying a single HV or a combined HV/LV pulse. Suspension cells from standard cultures (1×10^6^) were transfected with 4 µg pEGFP N1 vector using a single HV pulse (3 kV/cm, 400 µs; bold line) or a combined HV/LV pulse (3 kV/cm, 100 µs and 750 V/cm, 10 ms; gray area) applied by the ELPorator 1000 device. After 24 h, the transfection efficiency was determined by flow cytometry from the percentages of EGFP+ cells and from individual EGFP expression levels according to the MFI values. Light lines show the respective negative control. Viable cell recovery was assessed by trypan blue exclusion. Values given in individual columns are: EGFP+ cells as percentage of all cells; MFI x103; viable cell recovery x105. Data are representative for at least three comparable experiments done in triplicates. (0.15 MB TIF) [file pone.0009488.s002.tif]
